# Evaluating maize and soybean grain dry-down in the field with predictive algorithms and genotype-by-environment analysis

**DOI:** 10.1038/s41598-019-43653-1

**Published:** 2019-05-09

**Authors:** Rafael A. Martinez-Feria, Mark A. Licht, Raziel A. Ordóñez, Jerry L. Hatfield, Jeffrey A. Coulter, Sotirios V. Archontoulis

**Affiliations:** 1 Department of Agronomy, Iowa State University, Ames, IA 50011 USA; 2 USDA-ARS, National Laboratory for Agriculture and the Environment, Ames, IA 50011 USA; 3 Department of Agronomy and Plant Genetics, University of Minnesota, St. Paul, MN 55108 USA

**Keywords:** Plant physiology, Agroecology, Environmental impact

## Abstract

A delayed harvest of maize and soybean crops is associated with yield or revenue losses, whereas a premature harvest requires additional costs for artificial grain drying. Accurately predicting the ideal harvest date can increase profitability of US Midwest farms, but today’s predictive capacity is low. To fill this gap, we collected and analyzed time-series grain moisture datasets from field experiments in Iowa, Minnesota and North Dakota, US with various maize (n = 102) and soybean (n = 36) genotype-by-environment treatments. Our goal was to examine factors driving the post-maturity grain drying process, and develop scalable algorithms for decision-making. The algorithms evaluated are driven by changes in the grain equilibrium moisture content (function of air relative humidity and temperature) and require three input parameters: moisture content at physiological maturity, a drying coefficient and a power constant. Across independent genotypes and environments, the calibrated algorithms accurately predicted grain dry-down of maize (*r*^2^ = 0.79; root mean square error, RMSE = 1.8% grain moisture) and soybean field crops (*r*^2^ = 0.72; RMSE = 6.7% grain moisture). Evaluation of variance components and treatment effects revealed that genotypes, weather-years, and planting dates had little influence on the post-maturity drying coefficient, but significantly influenced grain moisture content at physiological maturity. Therefore, accurate implementation of the algorithms across environments would require estimating the initial grain moisture content, via modeling approaches or in-field measurements. Our work contributes new insights to understand the post-maturity grain dry-down and provides a robust and scalable predictive algorithm to forecast grain dry-down and ideal harvest dates across environments in the US Corn Belt.

## Introduction

As the growing season approaches its end and crops mature, farmers in the United States (US) Midwest turn their attention to monitoring maize (*Zea mays* L.) and soybean (*Glycine max* L. [Merr.]) grain moisture status in the field to establish appropriate harvest dates, a decision with important economic implications. The standard moisture content for grain marketing and safe storage^1^ ranges from 13 to 15.5%, depending on the type of grain and storage time. Harvesting grain below these thresholds results in lost revenue due to grain shrinkage^2^ and increases risk of yield losses caused by plant lodging, dropped grain, bird damage, and diseases^1,3–6^. Harvesting grain above these thresholds can also result in lost revenue due to buyer’s penalties in selling price or to the additional cost of artificially drying grain prior to marketing. In the northern US Midwest, the cost for artificial grain drying is the second or third largest expense in maize production after fertilizer or seed^7^. Each year farmers have to balance these tradeoffs, but currently only “rules of thumb” are available to estimate dry-down (e.g., 0.25 to 1.0% maize grain moisture loss per day^8,9^). These simplistic approaches are often imprecise. Therefore, developing and implementing data-driven tools that can predict grain dry-down in the field is needed to assist producers in decision-making.

The physiological process of grain moisture loss can be divided into two phases. The first phase takes place during grain filling, in which water is displaced by deposition of assimilates (e.g., starch, protein, oil). As the grain dry matter increases, percent grain moisture decreases. Studies in maize^10–14^ and soybean^15^ have shown that the rate of grain moisture loss during this phase is related to seed growth rate, maximum seed size and the duration of the grain filling period. Once grains reach their maximum dry matter accumulation, a stage called physiological maturity, transfer of fluids between the plant and seed ceases. At this point, kernel moisture of commercial maize hybrids normally ranges between 32 and 40%^11,12,16^, although this range can be broader for inbred lines (28–60%)^14^. In soybean, seed moisture at physiological maturity ranges between 55 and 65%^15,17,18^.

The second phase of grain moisture loss occurs after physiological maturity and is the focus of this work. During this phase, water is lost through physical evaporation from the grain surface^19^, a process primarily controlled by endosperm osmotic pressure and pericarp permeability^20,21^. Post-maturity grain dry-down typically follows a negative curvilinear response to days after physiological maturity^10,22^, and continues until grain moisture reaches equilibrium with the surrounding air^23^. This equilibrium moisture content depends on the properties of the drying material (e.g., grain type) and conditions of the air (i.e., temperature and relative humidity). Variable atmospheric conditions in the field cause the equilibrium point to be dynamic, thus making the scheduling of harvest operations challenging. With increasing weather variability in the US Midwest and other regions^24^, the need to predict grain dry-down in the field is becoming more pressing.

Most of the work on understanding and predicting seed moisture dynamics in both maize and soybean have focused on the grain fill period^14,25,26^, while the period of post-maturity grain dry-down remains much less explored^27–29^. Mathematical models to describe grain drying exist^30^, but mainly these have been used to predict grain moisture loss in controlled environments such as mechanical driers, and only a few have been adapted and tested for field conditions. Most recently, Piggot^31^ and Maiorano *et al*.^26^ adapted the Henderson and Perry^32^ equation to develop a mechanistic algorithm that simulates dry-down of maize grain in the field. The algorithm calculates post-maturity changes in grain moisture on a daily time-step, and requires as input the moisture content at physiological maturity (*M*_0_), a drying rate coefficient (*k*) and the equilibrium moisture content (*Me*). The latter is computed dynamically (see methods for details). While the algorithm has proven robust to simulate maize dry down, to our knowledge, this algorithm has not been implemented in soybean. Additionally, grain drying could also be affected by other weather variables, such as wind speed, air temperature and relative humidity, in ways that cannot be fully captured by changes in *Me*^26,33^. Thus, improved prediction ability can be achieved by including additional explanatory weather variables in the formulation of the algorithm. Finally, model parameters may differ among genotypes and environments^26^, but information regarding this is limited.

The scarcity of time-series field data to calibrate, test, and improve predictive approaches has been a major limitation for implementation. This gap is clearly reflected by the widespread inability of current crop models such as APSIM^34^, CropSyst^35^ or Hybrid-Maize^36^ to simulate post-maturity grain moisture dynamics, or the lack of stand-alone decision support tools for estimating harvest day. To fill this gap, we collected and analyzed time-series post-maturity grain moisture datasets from field experiments in Iowa, Minnesota and North Dakota, US with various maize (n = 102) and soybean (n = 36) genotype-by-environment treatments (Table 1). Our aim is to provide insight into the grain drying process, and develop scalable data-driven algorithms to assist farmers in decision-making. More specifically, our objectives were: (i) expand the application of the Henderson-Perry algorithm to soybean dry-down; (ii) test various explanatory-weather factors (temperature, relative humidity, wind speed, and their interactions) to improve prediction and explanatory power; and (iii) explore whether genotypes and their interaction with the environment affect model parameters to further inform implementation of the algorithm across environments.

**Table 1:** Summary of the data sources used to train and test the post-maturity grain dry-down algorithms for maize and soybean.

Site	Experimental treatments			Dataset	
	Year	Planting date	Genotype^†^	n	Split
**Maize**					
Ames, IA	2014	22-Apr, 9-May, 6-Jun	P0407 (104), P0987 (109), P1151 (111), P1365 (113)	12	Training
	2015	15-Apr, 13-May, 4-Jun	P0407 (104), P0987 (109), P1151 (111), P1365 (113)	12	Training
	2016	15-Apr, 10-May, 5-Jun	P0407 (104), P0987 (109), P1151 (111), P1365 (113)	12	Training
Crawfordsville, IA	2016	14-Apr, 9-May	P0636 (106), P1151 (111), P1365 (113)	6	Testing
	2017	13-Apr, 16-May	P0589 (105), P1197 (111), P1555 (115)	6	Testing
Kanawha, IA	2016	17-Apr, 18-May	P9526 (95), P0407 (104), P0987 (109)	6	Testing
	2017	17-Apr, 9-May	P0157 (101), P0589 (105), P1197 (111)	6	Testing
Fisher, MN	2016	2-May	P7332 (73), P7632 (76), P7958 (79), P8210 (82), P8761 (87)	5	Testing
	2017	29-Apr	P7332 (73), P7958 (79), P8210 (82)	3	Testing
Hunter, ND	2015	16-Apr	P8210 (82), P8673 (86)	2	Testing
	2016	30-Apr	P8673 (86), P8761 (87)	2	Testing
Kennedy, MN	2016	1-May	P7332 (73), P7632 (76), P7958 (79)	3	Testing
	2017	12-May	P7332 (73), P7632 (76), P7958 (79), P8210 (82), P8673 (86)	5	Testing
Larimore, ND	2016	21-Apr	P7332 (73), P7632 (76), P7958 (79), P8210 (82), P8761 (87)	5	Testing
	2017	6-May	P7332 (73), P7632 (76), P7958 (79), P8210 (82), P8673 (86), P8761 (87)	6	Testing
Red Lake Falls, MN	2016	4-May	P7332 (73), P7632 (76), P7958 (79)	3	Testing
Wannaska, MN	2016	8-May	P7332 (73), P7632 (76), P7958 (79)	3	Testing
Winger, MN	2017	7-May	P7332 (73), P7632 (76), P7958 (79), P8210 (82), P8673 (86)	5	Testing
**Soybean**					
Ames, IA	2014	6-May, 20-May, 10-Jun	P22T69 (2.2), P25T51 (2.5), 92Y75 (2.7), P35T58 (3.5)	12	Testing
	2015	6-May, 20-May, 10-Jun	P22T69 (2.2), P25T51 (2.5), 92Y75 (2.7), P35T58 (3.5)	12	Training
	2016	6-May, 19-May, 9-Jun	P22T69 (2.2), P25T51 (2.5), 92Y75 (2.7), P35T58 (3.5)	12	Training

Additional information is provided in the suppl. Table S1.

^†^Numbers between parentheses indicate relative maturity of the genotype.

## Data Sources and Analytical Procedures

### Grain moisture content data sources

Time-series maize and soybean grain moisture data were collected from a field experiment conducted in central Iowa. Additionally, maize grain moisture data were available for 10 additional sites in northern and southeast Iowa, northwestern Minnesota and eastern North Dakota. All of these sites have deep, fertile soils, and a humid continental climate. In total, the data encompassed 102 maize and 36 soybean genotype-by-environment treatments, which included measurements from 16 maize hybrids and 4 soybean cultivars. Table 1 shows a summary of the experimental treatments at each site. The experimental factors in the Ames (central Iowa) experiment, included four different genotypes per crop, three different planting dates per genotype and the experiments were repeated over three years (Table 1). Within a year, each experimental unit was replicated four times. The experiment was set up in a maize-soybean rotation with both crop phases present in each year. Maize was planted at 86,450 seeds ha^−1^ and soybean at 345,800 seeds ha^−1^ both at 76 cm row spacing. Three planting dates (early, mid, and late) were spaced at approximately at 25d intervals beginning in late April. The maize hybrids represented four relative maturities (104-day, 109-day, 111-day, and 113-day), and the soybean varieties represented four maturity groups (2.2, 2.5, 2.7, and 3.5). Soil fertility was managed according to university recommendations^37,38^. Maize ear and soybean pod samples were collected from late August to final (mechanical) harvest date at one-week intervals. Crop phenological stage was determined according to Abendroth *et al*.^39^ and Licht and Pedersen^40^ for maize and soybean, respectively. In the field, we collected two maize ears per plot and all the pods from a plant per plot. In the lab, we detached maize kernels from ears and soybean seeds from pods, weighed subsamples (100 g for maize and 10 g for soybean), and then placed in a forced-air oven at 105 °C until constant mass was achieved. The dry samples were placed in a desiccator with anhydrous calcium chloride for two hours to allow cooling of the sample and removal of the remaining moisture. The dry samples were weighed and percent moisture content was expressed on a wet basis (i.e., ratio of water mass in grain to total fresh grain mass).

The additional maize grain moisture datasets were collected between 2015 and 2017. These include measurements from two to six maize genotypes of differing relative maturities (73-day to 115-day) in each site-year. The Iowa datasets also included two planting date treatments (late-April and mid-May). Maize plots were managed following best practices for the region. After physiological maturity, ear samples were collected in intervals of 7 to 9d. Percent grain moisture was determined using AM-5200-A (Perten Instruments, Hägersten, Sweden) and GAC2500 (Dickey-John, Auburn, Ill. US) electronic meters. Detailed descriptions of the datasets are provided by Licht *et al*.^41^ for the Iowa sites, and by Coulter and Fore^42^ for the Minnesota and North Dakota sites.

### Weather data source

Weather data were obtained for each field site and included daily values of precipitation, temperature, mean relative humidity, and mean wind speed. In the Iowa experiments, the weather stations were located on-site and belong to the Iowa State University Soil Moisture (ISUSM) network. For the Minnesota and North Dakota sites, data was retrieved from the closest station belonging to the Automated Surface Observing System (ASOS) network (see Table S1 in the suppl. information). All of these data were accessed through the Iowa Environmental Mesonet web portal^43^.

### Dry-down model

The Henderson-Perry equation^32^ states that the change in grain moisture during a time interval is proportional to the difference between the grain moisture content (*M*; % wet basis) at time *x*, and the equilibrium moisture content (M_*e*_; %)

1 $$\frac{dM}{dx}=-\,k(M-{M}_{e})$$

where *k* is a proportionality drying coefficient. The equation is based on diffusion theory (i.e., Fick’s second law), which assumes that resistance to diffusion occurs mainly in a thin outer layer. In grains, this layer is often interpreted as the seed coat or pericarp, although the endosperm mass can also limit diffusion^30^. Piggot^31^ proposed to adapt this equation to simulate maize grain moisture loss in the field, and used two different *k* values for representing grain moisture loss before and after physiological maturity. The post-maturity phase also included an extra term to account for rewetting of the grain due to precipitation and heavy dew. Maiorano *et al*.^26^ argued that the Henderson-Perry equation was only adequate for the dry-down phase, and proposed an alternative model for the grain-filling phase. Here we only focus on the dry-down phase.

To further improve the model and expand its application to soybean, we modified the Henderson-Perry equation in two ways. First, following Page^44^ we generalized the power of *x* to a constant (*n*), so the amount of grain moisture loss on a given time-step *x* not only depends on the moisture content but also on the time elapsed since physiological maturity:

2 $$\frac{dM}{dx}=-\,k\cdot (M-{M}_{e})\cdot n\cdot {x}^{n-1}$$

Note that this expression is equal to the Henderson-Perry equation when *n* = 1. The power parameter provides additional flexibility in the model to fit the experimental data. Second, instead of using actual time (i.e., calendar days) as the *x*-independent variable, we use the accumulation of days scaled by how favorable weather conditions are for grain drying. The concept is similar to growing-degree days^45^ which are widely used to predict crop development. Finally, the integrated expression is:

3 $$M(x)=({M}_{0}-{M}_{e})\cdot {e}^{-k\cdot {x}^{n}}+{M}_{e}$$

where *M*_0_ is the grain moisture content at physiological maturity, which is R6 for maize^39^ and R6.5 for soybean^40^. The dynamic value for *Me* can be calculated using the following equation^23^:

4 $${M}_{e}={(\frac{\mathrm{ln}(1-\frac{RH}{100})}{-A(T+B)})}^{1/C}$$

where RH is relative humidity (%), *T* is daily mean temperature (°C), and *A*, *B* and *C* are constants specific to the drying material. Constants were parametrized as *A* = 0.0001557, *B* = 45.5, and *C* = 2 for maize derived from Thompson *et al*.^46^, and as *A* = 0.000729, *B* = 31.6, and *C* = 1.526 for soybean, according to Yang *et al*.^47^. These parametrizations produce results on a dry basis (i.e., ratio of water mass in grain to total dry grain mass), so they were converted to wet basis. Also, because of its dependence on weather, daily values of *Me* can vary greatly (see example in Suppl. Fig. S1), leading to unrealistically fast changes in grain moisture content. This was mitigated by using the 3-day moving average.

### Explanatory weather factors

In addition to *days* after physiological maturity, we explored three explanatory weather factors to scale the time-step: a relative humidity factor (*h*; Eq. 5), a temperature factor (*t*; Eq. 6) and a wind speed factor (*w*; Eq. 7):

5 $$h=\sum _{i=0}^{n}(1-\frac{R{H}_{i}}{100})$$

6 $$t=\sum _{i=0}^{n}(\frac{TMA{X}_{i}+TMI{N}_{i}}{2}-{T}_{base})\,\{\begin{array}{c}TMA{X}_{i} < {T}_{base}\,TMA{X}_{i}={T}_{base}\\ TMI{N}_{i} < {T}_{base}\,TMI{N}_{i}={T}_{base}\end{array}$$

7 $$w=\sum _{i=0}^{n}W{S}_{i}$$

where for the *i*^th^ day after physiological maturity, *RH* is mean relative humidity (%), *TMAX* and *TMIN* are maximum and minimum temperatures (°C), and *WS* is daily mean wind speed (m s^−1^). The *h* factor weights individual days by their drying potential (evaporative demand), with values ranging from 0 to 1. The *t* factor weights days by their temperature, equivalent to the second method described by McMaster and Wallace^45^ for calculating growing degree days, using a base temperature (*T*_*base*_) of 0 °C. Finally, the *w* factor weights days by how windy they are, with possible values ranging from 0 to infinity. Additional factors were computed by multiplying their two-way and three-way combinations (i.e., *h* × *t*, *h* × *w*, *t* × *w*, *h* × *w* × *t*). The default, non-scaled time series was reported as *day*.

### Model training

Data used for training of the maize models included all the experimental units from the Ames site (n = 36; Table 1), whereas the rest of the sites were used for testing. Because soybean data were only available for the Ames site, we used 2015 and 2016 data to train the soybean models, and 2014 for testing.

In model training, we estimated the *M*_0_, *k*, *and n* parameters for each model by fitting nonlinear regression equations for every weather factor to the integrated model (Eq. 3) with the nonlinear least squares function (*nls*) of the nonlinear and linear mixed effects package (*nlme*)^48^ in R statistical software^49^ (version 3.4.2). Test of significance for estimated parameters *M*_0_ and *k* was based on the null hypothesis that the parameter was equal to 0, whereas for *n* it was based on the null hypothesis that the parameter was equal to 1. Model fit to the training data was evaluated using the adjusted coefficient of determination (Adj. r^2^), root mean square error (RMSE), Akaike information criterion (AIC), Bayesian information criterion (BIC), and modeling efficiency (M_Eff_). The r^2^ reflects prediction ability, while M_Eff_ is a measure of improvement in model fit with respect to a simple mean, and for both of these the higher the value the better. The AIC and BIC are indices for model selection, while RMSE reflects model error. For the latter three indices, the lower the value the better.

### Genotype-by-environment analysis

We tested treatment effects on the dry-down process using the dataset from the Ames site (Table 1). Statistical *nls* optimizations were performed for every combination of crop, year, planting date and genotype at the central Iowa site to obtain model parameters for each experimental unit. Only the *M*_0_ and *k* parameters were estimated, whereas *n* was held constant. This is because previous analysis has shown strong correlation between *k* and *n* parameters, which prevents direct comparison of treatment effects^50^. Linear models of the effect of planting date, genotype, weather-year, and their interaction were fit independently to each dataset of *M*_0_ and *k* parameters for maize and soybean, using the PROC MIXED function in SAS 9.4 software^51^. From the resulting type-3 test of significance for fixed effects, the highest-level significant (α = 0.05) interactions or main effects were compared using the Tukey-Kramer adjustment. Additionally, variance components analysis was used to estimate the overall variability explained by genotype, weather-year, and planting date with the VARCOMP procedure in SAS using the restricted maximum likelihood method.

### Testing the implementation of the dry-down algorithms

The fitted models were evaluated by comparing predictions against the independent testing dataset (Table 1). Simulations were run using the differential version of the model (Eq. 2) on a daily-time step. The value of *k* and *n* parameters was set according to the results from model training, while *M*_0_ was set at the grain moisture content of the first measurement after physiological maturity. Simulation performance was assessed using the Adj. r^2^, RMSE, M_Eff_, in addition to the model bias (M_Bias_). The latter is a measure of model accuracy, and the closer the value to zero, the better. In addition, we fit simple linear regression equations of measured versus predicted values and calculated the slope as another measure of model accuracy, with a value closer to 1 being better. The equations for all of these metrics can be viewed in Archontoulis and Miguez^52^.

## Results

### Evaluating explanatory weather factors for use in the dry-down algorithm

To find the best predictor of grain dry-down in the field, we evaluated cumulative daily measurements (starting at physiological maturity) of relative humidity (*h*), temperature (*t*), wind speed (*w*) as well as their two-way and three-way combinations (i.e., *h* × *t*, *h* × *w*, *t* × *w*, *h* × *w* × *t*). By default, the dry-down algorithm uses days after physiological maturity (*day*) as the explanatory factor, which was also included in this study.

**Figure 1:**
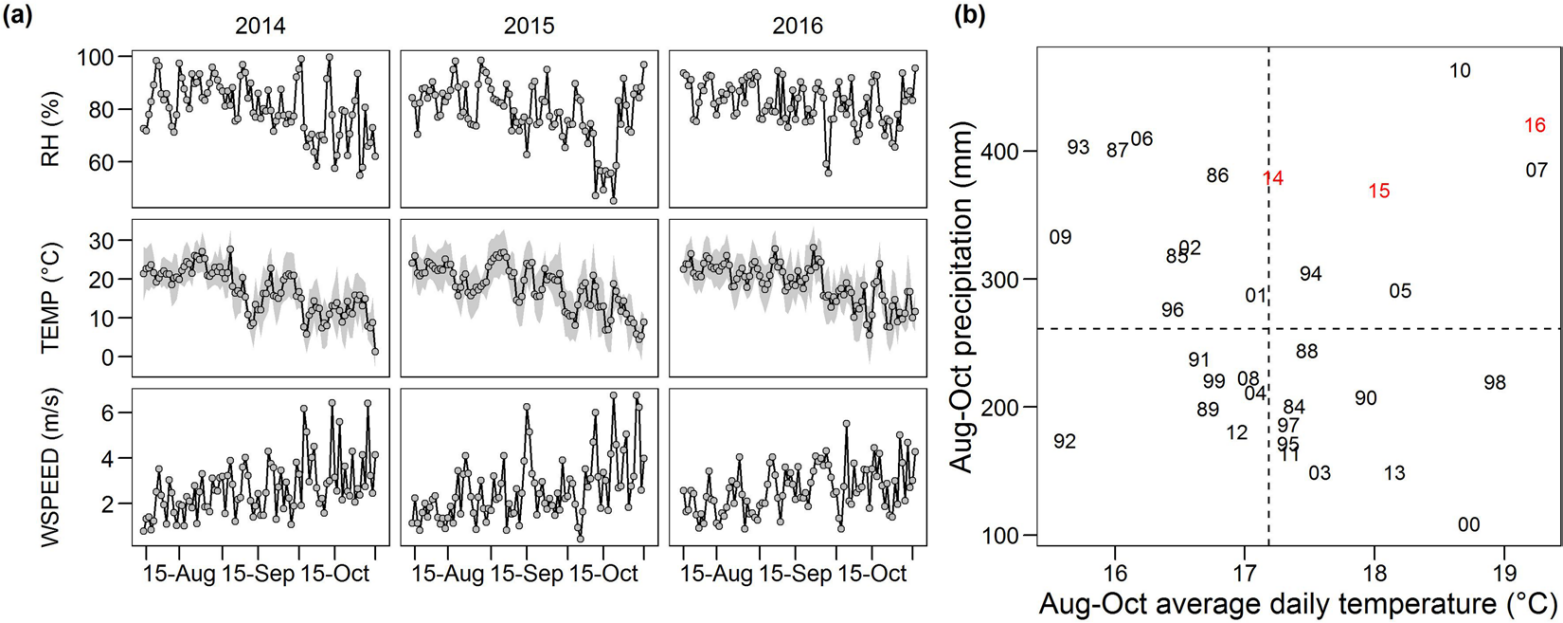
(**a**) Daily relative humidity (RH), temperature (TEMP), and wind speed (WSPEED) during the grain-fill and dry-down periods (August to October) at the Ames (central Iowa) site. Shaded area for temperature shows the spread between daily maximum and minimum temperatures. (**b**) Comparison of the experimental years (2014–2016; shown in red) to the 30-year climatic normal (1984–2013; in black). Crosshairs indicate mean precipitation (mm) and average daily mean temperature (°C) for the period.

Weather conditions during the late grain-filling and dry-down periods (August and October) in the Ames experiment tended to be warmer and wetter than the 30-year historical average (Fig. 1). Relative humidity generally oscillated around 80% (range: 45–100%) and wind speed oscillated around 3.8 m s^−1^ (range:1–7 m s^−1^).

Training data (Table 1) was used to estimate the moisture content at physiological maturity (*M*_0_), the drying rate coefficient (*k*), and the power constant (*n*) parameters. The *n* parameter in the maize models was not significantly different from 1 (p < 0.05; suppl. Table S2), indicating that the rate post-maturity grain moisture loss of maize grain is not directly dependent on time after physiological maturity. Therefore, we refitted the maize models by fixing *n* = 1. The estimated parameters for *M*_0_ and *n* were relatively stable within each crop, while estimates for the *k* parameter varied between crops (Table 2).

**Table 2:** Model parameter estimates (standard error in parenthesis) and test of significance of model fits to the data using days after maturity (*day*), humidity (*h*), temperature (*t*), wind speed (*w*) and their combinations as explanatory variables.

	*Mo*		*k*		*n* ^†^	
	(*%*)		(*unitless*)		(*unitless*)	
***Maize***						
*day* ^‡^	36.5 (0.451)	***	0.0622 (0.00277)	***	—	—
*h*	36.3 (0.420)	***	0.2720 (0.01180)	***	—	—
*t*	36.4 (0.525)	***	0.0038 (0.00019)	***	—	—
*w*	36.3 (0.452)	***	0.0215 (0.00098)	***	—	—
*h* *×* *t*	36.5 (0.463)	***	0.0171 (0.00079)	***	—	—
*h* *×* *w*	36.0 (0.427)	***	0.0888 (0.00408)	***	—	—
*t* *×* *w*	36.3 (0.518)	***	0.0013 (0.00007)	***	—	—
*h* *×* *t* *×* *w*	36.3 (0.465)	***	0.0057 (0.00027)	***	—	—
***Soybean***						
*day*	60.9 (1.27)	***	0.00404000 (0.0025900)	Ns	2.32 (0.263)	***
*h*	61.2 (1.29)	***	0.18300000 (0.0405000)	***	2.17 (0.247)	***
*t*	60.9 (1.38)	***	0.00000549 (0.0000079)	Ns	2.29 (0.269)	***
*w*	60.0 (1.32)	***	0.00021000 (0.0002290)	Ns	2.45 (0.310)	***
*h* *×* *t*	60.8 (1.27)	***	0.00013500 (0.0001410)	Ns	2.40 (0.278)	***
*h* *×* *w*	60.3 (1.36)	***	0.01470000 (0.0085600)	Ns	2.22 (0.295)	***
*t* *×* *w*	60.1 (1.50)	***	0.00000063 (0.0000012)	Ns	2.23 (0.290)	***
*h* *×* *t* *×* *w*	60.0 (1.39)	***	0.00001600 (0.0000231)	Ns	2.26 (0.290)	***

*M*_0_ = grain moisture content at physiological maturity; *k* = drying constant; *n* = power constant.

^†^H_0_: *n* = 1.

^‡^Significance codes: ns = (p > 0.05); *(0.05 > p > 0.01); **(0.01 > p > 0.001); ***(p < 0.001).

**Figure 2:**
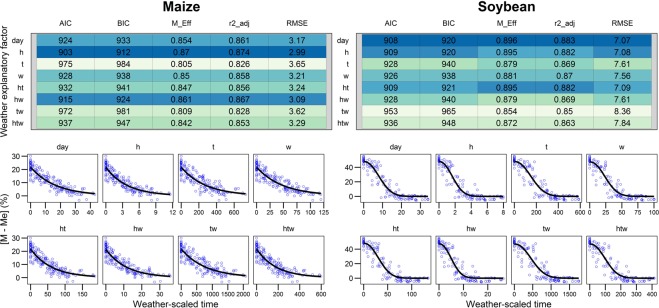
Parameterization of the dry-down models with various x-explanatory variables: days after physiological maturity (*day*), relative humidity (*h*), temperature (*t*), wind speed (*w*) and their combinations, using the training dataset (see Table 1). Model fit was evaluated using Akaike information criterion (AIC), Bayesian information criterion (BIC), modeling efficiency (M_Eff), adjusted coefficient of determination (r2_adj), and root mean square error (RMSE). Dark blue shading indicates better fit. Measured data are represented with open circles, while solid lines show fitted models.

The *day* model explained 86% of the temporal variation in the maize training data with an RMSE of 3.2%. Model fit was slightly improved by using *h* × *w* and *h*. All other factors decreased model fit (Fig. 2). Precision of model fit to soybean data was similar to maize, with the *day* model explaining 90% of the variation, albeit with greater error (RMSE = 7.1%). Performance of the model using *h* and *h* × *t* factors were essentially as good as *day*. All other weather factors decreased model fit (Fig. 2).

### Dissecting genotype-by-environment effects on dry-down

We used analysis of variance (ANOVA) to test whether model parameters (fitted to each experimental unit) were dependent on genotype, weather year or planting date during the three years of the experiment in Ames (Table 1). The ANOVA showed a significant effect (p < 0.05) of weather-year on the *M*_0_ parameter (grain moisture at physiological maturity) in maize, as well as a significant effect of the interaction of genotype and planting date on the *M*_0_ parameter in soybean (Table 3). In maize, the *M*_0_ was significantly greater in 2016 than in 2014, but not significantly different than in 2015. In soybean, the *M*_0_ was significantly higher in one genotype only between early- and mid-plantings. None of the experimental factors showed a significant effect in the *k* parameter (drying rate coefficient).

**Table 3:** Effect of genotype, weather-year and planting date on initial moisture content (*M*_*0*_) and drying coefficient (*k*) parameters of maize and soybean dry down algorithms, optimized for each experimental unit at Ames, Iowa.

		Maize		Soybean	
		*M*_*0*_	*K*	*M*_*0*_	*k*
***ANOVA Type-3 test of significance***		***(p > F)***			
Genotype (G)		0.334	0.592	0.733	0.630
Weather-year (Y)		0.003	0.237	0.134	0.424
Planting date (P)		0.107	0.513	0.743	0.342
G*Y		0.814	0.627	0.121	0.700
G*P		0.500	0.852	0.010	0.827
Y*P		0.373	0.407	0.167	0.575
***Effects on M0***					
**Maize**		**Soybean**			
			**Planting date**		
**Weather-year**	**M0 (%)**	**Genotype Rel. Mat**.	**Early**	**Mid**	**Late**
			**M0 (%)**		
**2014**	34.2 A	**2.2**	62.5 ab	63.9 ab	63.4 ab
**2015**	35.1 Ab	**2.5**	62.3 ab	64.4 ab	62.4 ab
**2016**	39.6 B	**2.7**	60.5 ab	64.4 ab	61.4 ab
		**3.5**	65.9 b	57.3 a	66.1 ab

**Figure 3:**
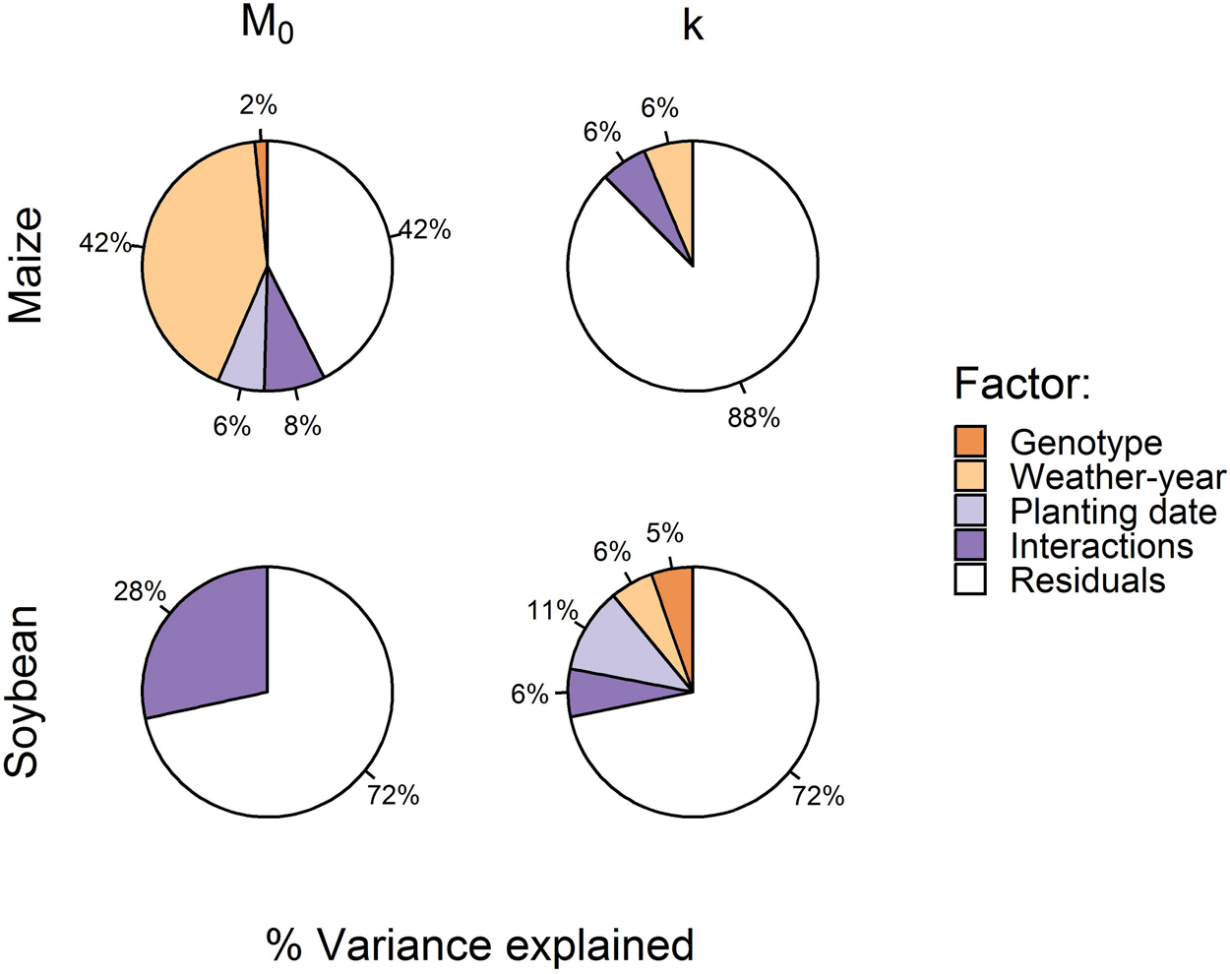
Variance components (%) associated with genotype, weather-year, planting date, and their two-way interactions for estimates of grain moisture at physiological maturity (*M*_*0*_) and drying constant (*k*) parameters from dry down models fitted to experimental units.

Variance component analysis revealed that the largest share of the variance for the *M*_0_ and *k* parameters could be attributed to the experimental error, while the rest could be explained by genotype, weather-year, planting date, or their interactions (Fig. 3). In maize, variance in *M*_0_ was largely driven by weather-year (42%), while the combination of genotype and weather-year played a small role (8%). Little variation (10%) in *k* parameters could be explained by experimental factors, which is consistent with the ANOVA results. In soybean, the picture was more complex. The interactions of experimental factors explained most of the non-error variance in *M*_0_ estimates (28%), while for *k*, genotype, weather-year, planting date, and their interactions together explained roughly equal amounts of the variance in parameter estimates (6–11%). In summary, experimental factors had some influence on values of *M*_0_, but not on *k*.

### Testing the prediction of the dry-down algorithms

**Figure 4:**
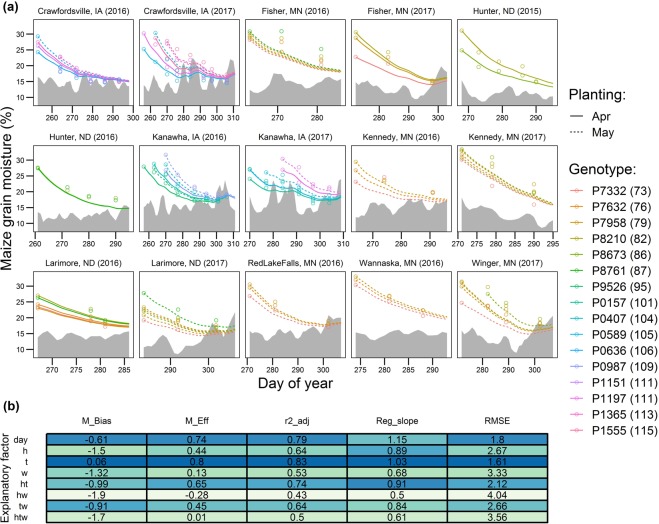
(**a**) Implementation of the maize grain-dry down algorithm across independent sites, planting dates and genotypes (testing dataset; Table 1). Solid lines represent simulation with the *day* algorithm, round symbols represent the measured data, and shaded area represents the 3-day moving average equilibrium moisture content (*Me*). Numbers within parentheses next to the genotype name indicate hybrid relative maturity. (**b**) Model fit among all the explored algorithms are compared using the model bias (M_Bias), modeling efficiency (M_Eff), adjusted coefficient of determination (r^2^_adj), slope of the regression of measured vs predicted (Reg_slope) and root mean square error (RMSE). Dark blue shading indicates better fit.

The calibrated maize models were able to explain 43 to 83% of the variation in the testing dataset (Fig. 4; Table 1), with a slight tendency to under-predict dry-down by −1.9 to 0.06% moisture. Only the *t*, *day* and *h* × *t* algorithms offered substantial modeling improvements compared to a simple mean (M_Eff_ > 50%). The *day* algorithm satisfactorily simulated grain moisture across most genotype-by-environment scenarios, capturing a large portion of the variation in post-maturity maize grain moisture (Adj. r^2^ = 0.77), with good efficiency (M_Eff_ = 73%), small error (RMSE = 1.9%) and little bias (M_Bias_ = −0.6). Performance of the *day* algorithm was slightly surpassed by the *t* algorithm. Based on computed statistical indices, the maize models ranked (best to worst): *t* > *day* > *h* × *t* > *t* × *w ~h* > *w* > *h* *×* *t* *×* *w* > *h* *×* *t* *×* *w* (Fig. 4).

**Figure 5:**
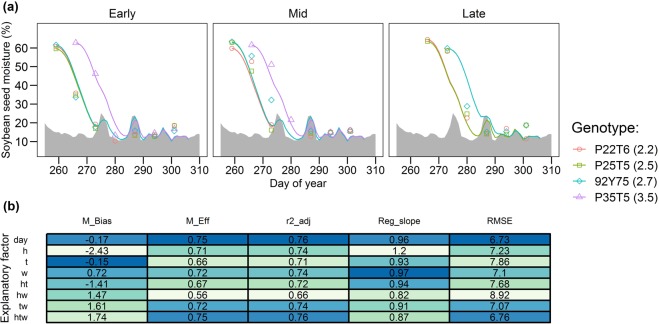
(**a**) Implementation of the soybean grain dry-down across independent environmental conditions (Early, mid and late planting dates in 2014; testing dataset in Table 1). Solid lines represent simulation with the *day* algorithm, symbols represent the measured data, and shaded area represents the 3-day moving average equilibrium moisture content (*Me*). Numbers within parentheses next to the genotype name indicate cultivar relative maturity. (**b**) Model fit among all the explored algorithms is compared using the model bias (M_Bias), modeling efficiency (M_Eff), adjusted coefficient of determination (r2_adj), slope of the regression of measured vs predicted (Reg_slope) and root mean square error (RMSE). Dark blue shading indicates better fit.

The calibrated soybean models explained 66 to 72% of the variation in the testing dataset (Fig. 5; Table 1) and fit to the testing data was similar to the training data (Fig. 2). All of the soybean models performed substantially better than a simple mean (M_Eff_ > 50%). Similar than in maize, the soybean dry-down was captured well by *day* algorithm, with good precision (Adj. r^2^ = 0.76), efficiency (M_Eff_ = 74%), acceptable error (RMSE = 6.7% grain moisture) and little bias (M_Bias_ = −0.17; Fig. 5). The *h* *×* *t* *×* *w* algorithm also had similar precision but showed a positive bias (M_Bias_ = 1.74%) meaning that tended to overestimate moisture content. The soybean models ranked (best to worst): *day* > *h* *×* *t* *×* *w* > *w* > *t* *×* *w* > *t* > *h* > *h* *×* *t* > *h* *×* *w* (Fig. 5).

## Discussion

Due to weather variability and logistic constraints, maize and soybean crops in temperate regions are often harvested at moisture contents above or below the ideal levels required for grain marketing and storage, which leads to additional operation costs. Currently, US Midwest farmers and crop consultants generally estimate harvest timing with ‘rules of thumb’ that assume linear rates of dry-down^8,9^. However, this approach cannot be reliably extrapolated across environments because it does not account for fluctuations in weather conditions (see example in Suppl. Fig. S2). Here, we parameterized and tested scalable data-driven algorithms to provide a more mechanistic prediction of grain dry-down in Midwestern fields. Coupling our algorithms with forecasted weather and economic models could allow decision makers to reliably estimate optimal harvest dates that minimize operational costs and risks, thus increasing profitability.

Previous work in maize^31^ evaluated the dry-down process using a non-linear exponential-decay algorithm running on a daily time-step (herein the *day* algorithm). It is important to note that the *day* algorithm already considers relative humidity and temperature effects on dry-down via the calculation of the equilibrium grain moisture content (*Me*; see Eq. 4 and Suppl. Fig. S1). Temperature is related to drying potential of the air, mainly through changes in the vapor pressure deficit, whereas air relative humidity controls the rate of water vapor transport from the grain surface to the surrounding air^53,54^. Other variables such as atmospheric pressure and airflow are also known to affect drying^55,56^, but these are not accounted in the computation of *Me*.

In grain driers *Me* is relatively constant, but in the field this value is dynamic^26,31^. Because *Me* is calculated based on data from weather stations, which are usually located outside the field, these conditions may be different from the micro-environment that grains experience during drying (i.e., protected by husks or pods). Therefore, a sudden change in weather (e.g., relative humidity) that can cause a large change in *Me* (Suppl. Fig. S1), might not translate to such a sudden change in moisture content. Here, we solved this problem by using a 3-day moving average, which helped stabilize *Me* and improve model fit. Although further smoothing could be achieved by using longer averaging periods (e.g., 5–7 days), this may result in the overestimation of *Me* in days with high drying potential and hence predict slower drying. Including a rewetting coefficient in the dry-down algorithm was considered in a previous study to account for the effect of precipitation and heavy dew^31^, but this was at the cost of additional input parameters and data requirements^26^. In fact, the effects of precipitation and dew are already partially captured by the relative humidity data because these events essentially occur when air is completely saturated (i.e., relative humidity ~100%). High relative humidity leads to an increase in *Me*, and when *Me* becomes greater than the grain moisture content, the change is then positive resulting in rewetting of the grain (see Figs. 4 and 5).

Here, we evaluated new algorithms with additional weather factors, aiming to improve predictive ability. For instance, the *t* algorithm considers additional temperature effects on dry-down, because the algorithm is run on a thermal time-step (i.e., growing degree days) instead than on a daily time-step. This is similar for the algorithms *h* (relative humidity-scaled time), *w* (wind speed-scaled time) and their combinations (see methods for details). Our analysis suggests that the *day* model is very robust, as it captured a large portion of the variation in grain moisture in training and testing datasets (Figs. 2, 4 and 5). While some improvements in prediction ability were achieved with *h* or *t* algorithms, their performance was not consistent across training and testing datasets or sites. The *t* algorithm performed better in Minnesota and North Dakota, while *h* performed better in Iowa. This could indicate potential overfitting when including additional weather explanatory factors. Besides, the improvements in model performance were relatively small, suggesting that the mechanisms of grain dry-down are already captured well by dynamic changes in *Me*. We provided calibrated parameters for all models (Table 2) for users to choose based on data availability and domain.

By having a dataset (n = 36) that captures genotype, weather-years, and planting date effects on model parameters for each crop (Ames experiment in Table 1), we were able to examine the relative importance of each of these factors and their interactions in the dry-down process (Table 3 and Fig. 3). We found that the studied factors affected grain moisture at physiological maturity (*M*_0_) but not the drying coefficient (*k*). Changes in *M*_0_ are mostly driven by source-sink dynamics during grain filling. Studies in cereal crops have shown that while physiological maturity is normally reached at about 35%^12^, stresses during this period can cause decoupling of the moisture and dry matter dynamics in the grain, mainly due to the premature cessation of dry matter accumulation^11–14,57,58^. Hence, physiological maturity is reached with higher grain moisture levels in crops under stresses such as terminal drought, heat shock, late-season diseases, or other source-sink restrictions. In the Ames (central Iowa) study, maize *M*_0_ was significantly higher in 2016 (Table 3). That year had much warmer than average conditions during late grain fill (i.e., August and September; Fig. 1), implying that the higher *M*_0_ could have been related to late-season heat stress. Genotypic variation in *M*_0_ has been also documented across inbred maize lines^14^, but differences are generally less significant among commercial hybrids^11^. Here, we found that maize *M*_0_ was not significantly different among the four genotypes in the Ames experiment (Table 3).

By comparison, soybean seed moisture and dry matter dynamics during grain fill have been shown to be less sensitive to stresses^13,59,60^ or genotypic traits such as seed size^15^. In our study, however, the 3.5 relative maturity cultivar had significantly lower *M*_0_ in the mid-planting dates (Table 3) in two out of the three years. Yet, the reason for this behavior is not entirely clear. Since the 3.5 relative maturity cultivar has a longer growth cycle than what is recommended for central Iowa^61^, it is possible that dry matter accumulation in the seed could had been constrained by a shortened duration of grain filling in the late plantings due to the occurrence of killing frosts prior to crop physiological maturity. However, this does not explain the higher *M*_0_ in the early plantings. More investigation to address this question is needed.

The post-maturity drying coefficient (*k*) is essentially a proportionality constant. For example, in the maize *day* algorithm *k* = 0.062 (Table 2), meaning that at every time-step the grain loses or gains 6.2% of the remaining available moisture (i.e. the difference between the moisture content of the grain and the point of equilibrium). In other words, the *k* parameter could also be thought as a “resistance to diffusion” coefficient. Because environmental factors are already captured by changes in the equilibrium point, it is expected that neither weather-year nor planting date would significantly affect *k*. However, crop genotypic traits could influence resistance to diffusion (i.e., *k*). For maize these include: husk number, tightness, length and senescence, ear length and angle, and number of grain per rows^4^. However, here we did not find significant effects of genotypes on *k* (Table 1). In contrast, Yang *et al*.^27^ detected significant differences among maize hybrids in a breeding program, but in that study grain samples were collected 45 days after silking, irrespective of whether the plants had achieved physiological maturity. Similarly, Poeta *et al*.^15^ used a single quadratic seed desiccation model to describe soybean grain moisture changes during the grain-fill and dry-down phases (R5 to R8). As noted earlier, moisture loss before and after maturity are driven by distinct processes, and a failure to distinguish between the two phases may lead to confounding results because the traits controlling grain fill rate are different from those controlling post-maturity moisture loss.

Soybean genotypic traits such as thickness of the pod wall and senescence^62^, or seed characteristics^63,64^, can influence the drying rate *k* coefficient. In a laboratory experiment, Giner *et al*.^64^ found differences among 25 Argentinian soybean varieties and showed that drying times were related to seed size, with larger seeds having longer drying times (i.e., lower *k*). In these controlled environment assays, drying of soybean followed a clear exponential-decay trajectory. However, this was not the case with our field data, where drying rates changed as drying progressed (see s-shaped pattern in Fig. 2). Explicitly including the power parameter (*n*) in the soybean algorithm helped to deal with this non-constant drying rate. While it has been previously argued that the *n* parameter does not have a clear biological interpretation in the drying process^50^, in soybean this may possibly be related to processes such as grain de-greening and pod senescence that occur alongside grain dry-down^17,65,66^. On the other hand, the *n* parameter in maize was not statistically different than 1 (suppl. Table S2), meaning that the amount of moisture loss of maize grains is not dependent on time.

In light of these results, important implications arise for developing a robust parameterization of the dry-down algorithm for implementation in existing crop models and for development of stand-alone tools to forecast moisture loss and harvest date across environments. Among the parameters in the dry-down algorithm, we found that *M*_0_ is the most sensitive (Table 3) meaning that this parameter should be estimated for specific situations. By using the moisture content of the first sample in the implementations we show that if *M*_0_ is known, dry-down can be predicted well for a range of genotypes and environmental conditions (Figs. 4 and 5). For crop simulation models, this means that the post-maturity dry-down algorithm needs to be coupled to a grain-fill moisture algorithm to predict *M*_0_, like the one proposed by Maiorano *et al*.^26^. In stand-alone decision support tools, field-estimated *M*_0_ values at a given date could be supplied by farmers, perhaps based on field readings obtained by electronic moisture meters^27^.

The fact that we did not find significant differences in the *k* coefficient across genotypes, weather-years and planting dates suggest that a species-specific *k* value for maize may be sufficient to simulate post-maturity grain moisture in commercial genotypes within the US Midwest (Fig. 4). This agrees with Maiorano *et al*.^26^, who showed that use of a single *k* value resulted in good model fit to maize grain moisture measurements across 11 genotypes in 9 weather years. However, it should be noted that these findings are based on evaluation of a few commercial genotypes, well adapted to regional production conditions (Table 1). It is likely that differences could be more discernable in populations with wider genotypic variation, such as in breeding programs^14,27^. Evidence of this variation is that modern maize genotypes have more concentrated and shorter periods of grain dry-down than older ones^27^. In the case of soybean, further work is needed to evaluate extrapolation of the algorithms beyond the one site for which data was available in this study.

Implementation of the dry-down algorithms may be constrained because relative humidity data (needed to compute the equilibrium moisture content) are not universally available from weather databases and forecasting systems. In the absence of direct relative humidity measurements, crop models such as APSIM and CropSyst simulate water exchange between the crop canopy and the atmosphere by assuming that the daily average dew point in humid and sub-humid climates is near the daily minimum temperature^67,68^. Under this assumption, daily relative humidity can be estimated from maximum and minimum air temperatures (see suppl. information S3 for details and examples). However, researchers should be aware that this approach may not be applicable in environments where relative humidity and temperature conditions are very different during dry-down (i.e., arid locations). Therefore, data availability constraints, as well as the tradeoffs with predictive ability, need to be taken in consideration when developing, adapting, and implementing these algorithms into modeling platforms and decision support tools.

## Conclusion

We parameterized scalable post-maturity grain dry-down algorithms for maize and soybean crops to aid harvest date decisions, aiming to increase profitability of US Midwest farms. The algorithms are driven by changes in the grain equilibrium moisture content (function of air relative humidity and temperature), and three input parameters: moisture content at physiological maturity, a drying coefficient, and a power constant. As opposed to rules of thumb that assume a linear rate of moisture decline, this approach allows for mechanistic predictions across environments. Our work advances previous efforts to predict maize dry-down in the field and proposes a new algorithm for predicting soybean dry-down. Analysis of comprehensive time-series datasets revealed that maize and soybean genotype-by-environment interactions had little influence on the post-maturity drying rate coefficient, but significantly influenced grain moisture content at physiological maturity. Thus, accurate implementation of the algorithms across environments would require estimating the initial grain moisture content, via modeling approaches or in-field measurements.

## Supplementary information

Supplementary Information

## Data Availability

The datasets collected and analyzed are available from the corresponding author on request.
