# Supplementary material for: Evaluating maize and soybean grain dry-down in the field with predictive algorithms and genotype-by-environment analysis
Source: Sci Rep. 2019 May 9;9:7167. doi: 10.1038/s41598-019-43653-1 (PMC6509253; doi:10.1038/s41598-019-43653-1)
Supplement: Supplementary file 1 — Supplementary Information [file 41598_2019_43653_MOESM1_ESM.docx]

**Evaluating maize and soybean grain dry-down in the field with predictive algorithms and genotype-by-environment analysis**

Rafael A. Martinez-Feria^a^, Mark A. Licht^a^, Raziel A. Ordóñez^a^, Jerry L. Hatfield^b^, Jeffrey A. Coulter^c^ and Sotirios V. Archontoulis^a*^

^a^Department of Agronomy, Iowa State University, Ames, IA 50011-1010, USA.

^b^USDA-ARS, National Laboratory for Agriculture and the Environment, Ames, IA 50011, USA.

^C^Department of Agronomy and Plant Genetics, University of Minnesota, St. Paul, MN 55108, USA.

^*^Corresponding author: [sarchont@iastate.edu](mailto:sarchont@iastate.edu)

**Supplemental information**

**Supplemental Table S1.** Location of the field sites and the corresponding weather stations from which daily weather data was retrieved (data source: Iowa Environmental Mesonet (<https://mesonet.agron.iastate.edu/>)

| ***Site*** | |  | ***Weather Station*** | |
| --- | --- | --- | --- | --- |
| ***Name*** | ***Coordinates*** |  | ***ID*** | ***Network*** |
| Ames, IA | 42.0,-93.7 |  | BOOI4 | ISUSM |
| Crawfordsville, IA | 41.2,-91.7 |  | CRFI4 |  |
| Kanawha, IA | 42.9,-93.8 |  | KNAI4 |  |
| Fisher, MN | 47.8,-96.8 |  | CKN | ASOS |
| Hunter, ND | 47.2,-97.2 |  | FAR |  |
| Kennedy, MN | 48.6,-96.8 |  | HCO |  |
| Larimore, ND | 47.9,-97.7 |  | RDR |  |
| Red Lake Falls, MN | 47.8,-96.3 |  | TVF |  |
| Wannaska, MN | 48.7,-95.9 |  | ROX |  |
| Winger, MN | 47.6,-96.1 |  | FSE |  |

**Supplemental Table S2.** Non-linear model parameter estimates (standard error in parenthesis) and test of significance of model fits to the data using days after maturity (*day*), humidity (*h*), temperature (*t*), wind speed (*w*) and their combinations as explanatory variables. *M_0_* = grain moisture content at physiological maturity; *k* = drying constant; *n* = power constant.

|  |  | ***M_0_*** | | ***k*** | | ***n****^†^* | |
| --- | --- | --- | --- | --- | --- | --- | --- |
|  |  | *(%)* | | *(unitless)* | | *(unitless)* | |
| ***Maize*** | |  |  |  |  |  |  |
|  | ***day***^‡^ | 36.4 (0.562) | *** | 0.0463 (0.01260) | *** | 1.090 (0.0911) | Ns |
|  | ***h*** | 36.5 (0.558) | *** | 0.2890 (0.03490) | *** | 0.951 (0.0766) | Ns |
|  | ***t*** | 36.5 (0.612) | *** | 0.0017 (0.00102) | Ns | 1.120 (0.1010) | Ns |
|  | ***w*** | 36.4 (0.615) | *** | 0.0211 (0.00775) | ** | 1.010 (0.0912) | Ns |
|  | ***h****×****t*** | 36.4 (0.547) | *** | 0.0118 (0.00435) | ** | 1.070 (0.0851) | Ns |
|  | ***h****×****w*** | 36.5 (0.603) | *** | 0.1280 (0.02520) | *** | 0.863 (0.0750) | Ns |
|  | ***t****×****w*** | 36.5 (0.665) | *** | 0.0012 (0.00080) | Ns | 1.000 (0.0970) | Ns |
|  | ***h****×****t****×****w*** | 36.4 (0.594) | *** | 0.0069 (0.00306) | * | 0.945 (0.0811) | Ns |
| ***Soybean*** | |  |  |  |  |  |  |
|  | ***day*** | 60.9 (1.27) | *** | 0.00404000 (0.0025900) | Ns | 2.32 (0.263) | *** |
|  | ***h*** | 61.2 (1.29) | *** | 0.18300000 (0.0405000) | *** | 2.17 (0.247) | *** |
|  | ***t*** | 60.9 (1.38) | *** | 0.00000549 (0.0000079) | Ns | 2.29 (0.269) | *** |
|  | ***w*** | 60.0 (1.32) | *** | 0.00021000 (0.0002290) | Ns | 2.45 (0.310) | *** |
|  | ***h****×****t*** | 60.8 (1.27) | *** | 0.00013500 (0.0001410) | Ns | 2.40 (0.278) | *** |
|  | ***h****×****w*** | 60.3 (1.36) | *** | 0.01470000 (0.0085600) | Ns | 2.22 (0.295) | *** |
|  | ***t****×****w*** | 60.1 (1.50) | *** | 0.00000063 (0.0000012) | Ns | 2.23 (0.290) | *** |
|  | ***h****×****t****×****w*** | 60.0 (1.39) | *** | 0.00001600 (0.0000231) | Ns | 2.26 (0.290) | *** |
| ^†^ H_0_: *n* = 1  ^‡^ Significance codes: Ns = (p > 0.05); * = (0.05 > p > 0.01); ** = (0.01 > p > 0.001); *** = (p < 0.001) | | | | | | | |


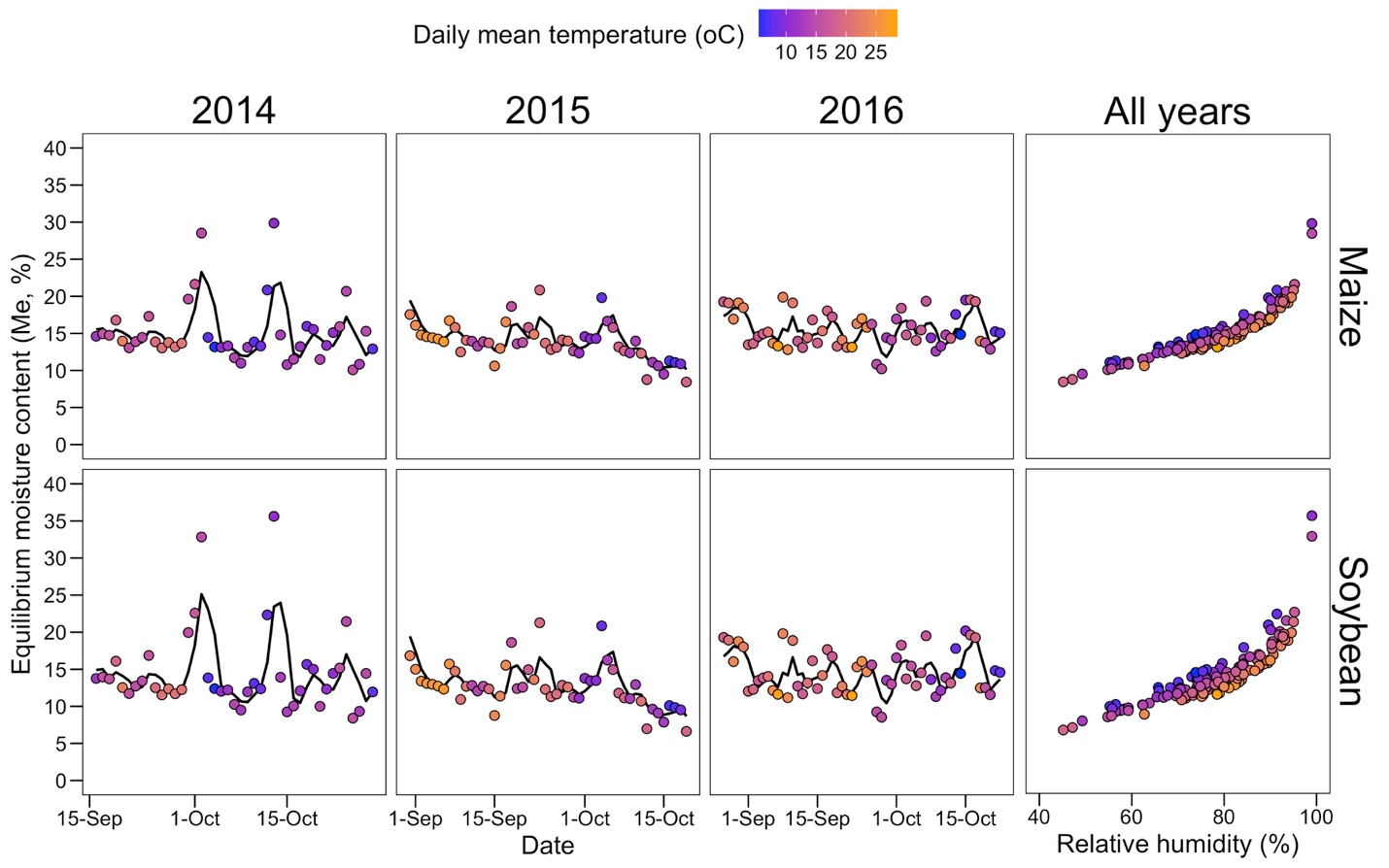


**Supplemental Figure S1**. Maize and soybean equilibrium moisture content (M*e*, %) during the grain dry down period in central Iowa in 2014, 2015, and 2016. Filled circles represent daily values of *Me*, with color ramp to indicate colder (blue) and warmer (orange) daily mean temperatures. Solid line represents the 3-day moving average of *Me.* Top and bottom right-most panels show the relationship of maize and soybean daily values of *Me* as affected by relative humidity (%) and mean temperature (°C).

**
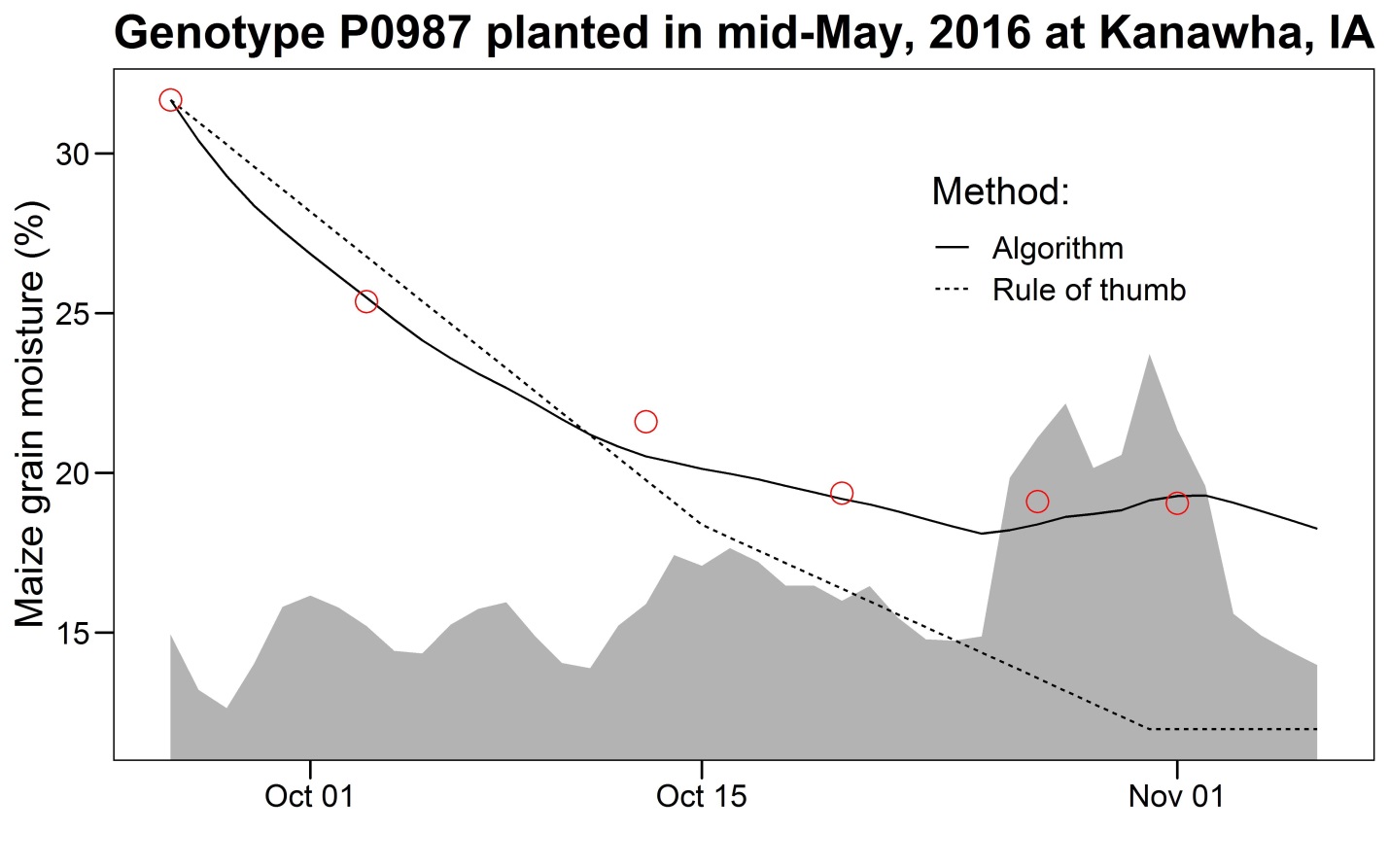
**

**Supplemental Figure S2.** Example comparing the prediction of the maize *day* dry-down algorithm versus the extension ‘rule of thumb’, which assumes 0.7 % of grain moisture loss per day the first 20 days after physiological maturity, and 0.4 % per day the thereafter (no drying after November 1^st^; see <https://crops.extension.iastate.edu/cropnews/2017/09/corn-grain-dry-down-field-maturity-harvest>). Lines represent predictions, round symbols represent the measured data, and shaded area represents the 3-day moving average equilibrium grain moisture content (*Me*).

**Supplemental information S3: Estimating daily mean relative humidity from minimum and maximum temperatures**

Relative humidity (RH; %) is the ratio of the partial pressure of water vapor in the air to the saturation vapor pressure of water (SVP) at a given temperature (*T*). Under typical atmospheric conditions, SVP can be computed using the following equation:

$SVP=0.61078\cdot e^{\frac{17.269 \cdot T}{273.3 + T}}$ (S1.1)

Estimation of daily mean RH begins by assuming that this value is well represented by the weighted average of the minimum and maximum RH for a given day:

$\frac{RH_{i}}{100}=\cdot\frac{\left( w_{1}\cdot{RH}_{Max}+ w_{2}\cdot{RH}_{Min} \right)}{2}$ (S1.2)

where *w_1_* and *w_2_* are weights. If we assume that the daily average dew point (i.e. temperature where air would be saturated) in humid and sub-humid climates is reasonably approximated by the daily minimum temperature, then we can substitute the RH terms such that:

$RH_{mean}=50\cdot\left( w_{1}\cdot\frac{{SVP}_{Tmin}}{{SVP}_{Tmin}}+ w_{2}\cdot\frac{{SVP}_{Tmin}}{{SVP}_{Tmax}} \right)= 50\cdot w_{1}+ 50\cdot w_{2}\cdot\frac{{SVP}_{Tmin}}{{SVP}_{Tmax}}$ (S1.3)

The first term represents the ${RH}_{Max}$ (i.e. 100%) when the air temperature reaches the dew point (e.g. at night), while the second term represents ${RH}_{Min}$ where temperature reaches its peak (e.g. afternoon). Finally, we can simplify the equation by substituting the product of the weights and 50 by regression coefficients:

$RH_{mean}=\beta_{0}+ \beta_{1}\cdot\frac{{SVP}_{Tmin}}{{SVP}_{Tmax}}$ (S1.4)

We fitted this linear model to daily weather measurements collected from 2013-2017 at 24 stations across Iowa (source: Iowa Environmental Mesonet; Fig. S3(a)), resulting in $\beta_{0}=$ 59 and $\beta_{1}=$44 (Fig. S3b). This approximation captured nearly 58% of the variation in daily RH measurements, although it tended to overestimate days with low RH (i.e. >60%; Fig. S3(b)).

Running the algorithms with estimated values of RH led to a slight underestimation of grain drying during days with low RH, although this only slightly affected simulation of dry-down. Note that the fit of the *day* algorithms (Figs S4-S5) was comparable to when running the model with actual RH data (Figs 4-5 in main text), suggesting that this approach may be a feasible alternative for when RH observation are missing.


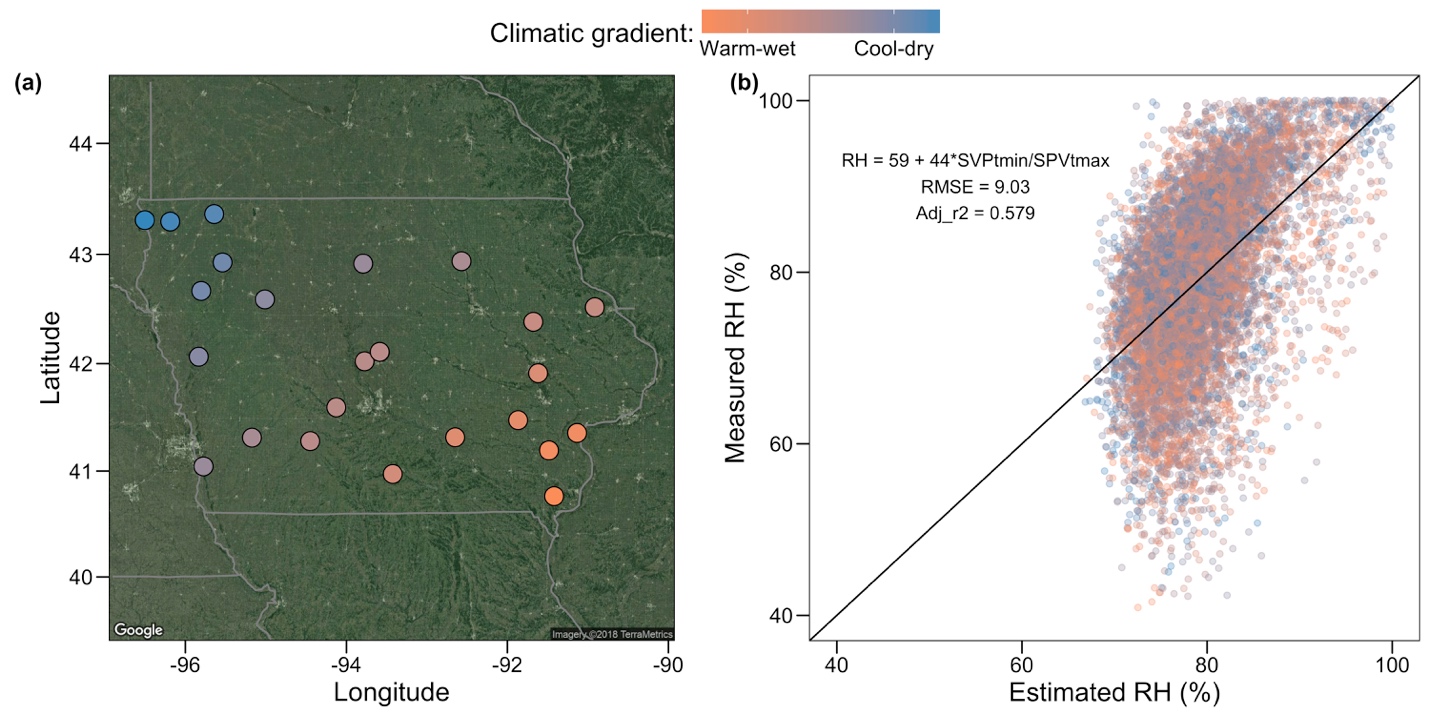


**Supplemental Figure S3**. (a) Location of the 24 stations where daily weather was used to fit regression constants for estimating relative humidity (RH). (b) Comparison of measured vs. estimated values. Satellite imagery source: Google, TerraMetrics.


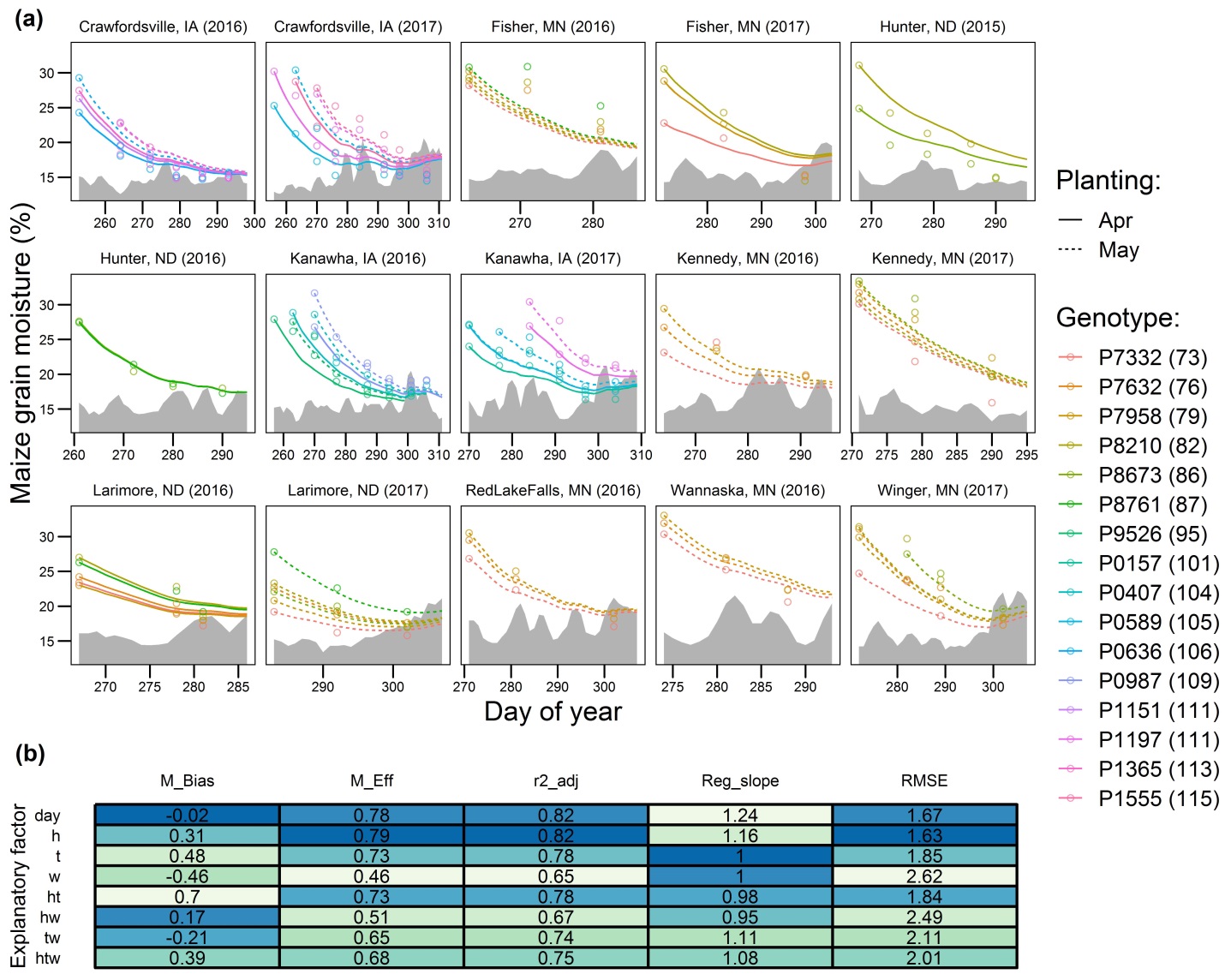


**Supplemental Figure S4**. (a) Implementation of the maize grain-dry down algorithm across independent sites, planting dates and genotypes (testing dataset; Table 1) using values of relative humidity estimated from daily minimum and maximum temperatures. Solid lines represent simulation with the *day* algorithm, round symbols represent the measured data, and shaded area represents the 3-day moving average equilibrium moisture content (*Me*). Numbers within parentheses next to the genotype name indicate hybrid relative maturity. (b) Model fit among all the explored algorithms are compared using the model bias (M_Bias), modeling efficiency (M_Eff), adjusted coefficient of determination (r2_adj), slope of the regression of measured vs predicted (Reg_slope) and root mean square error (RMSE). Dark blue shading indicates better fit.


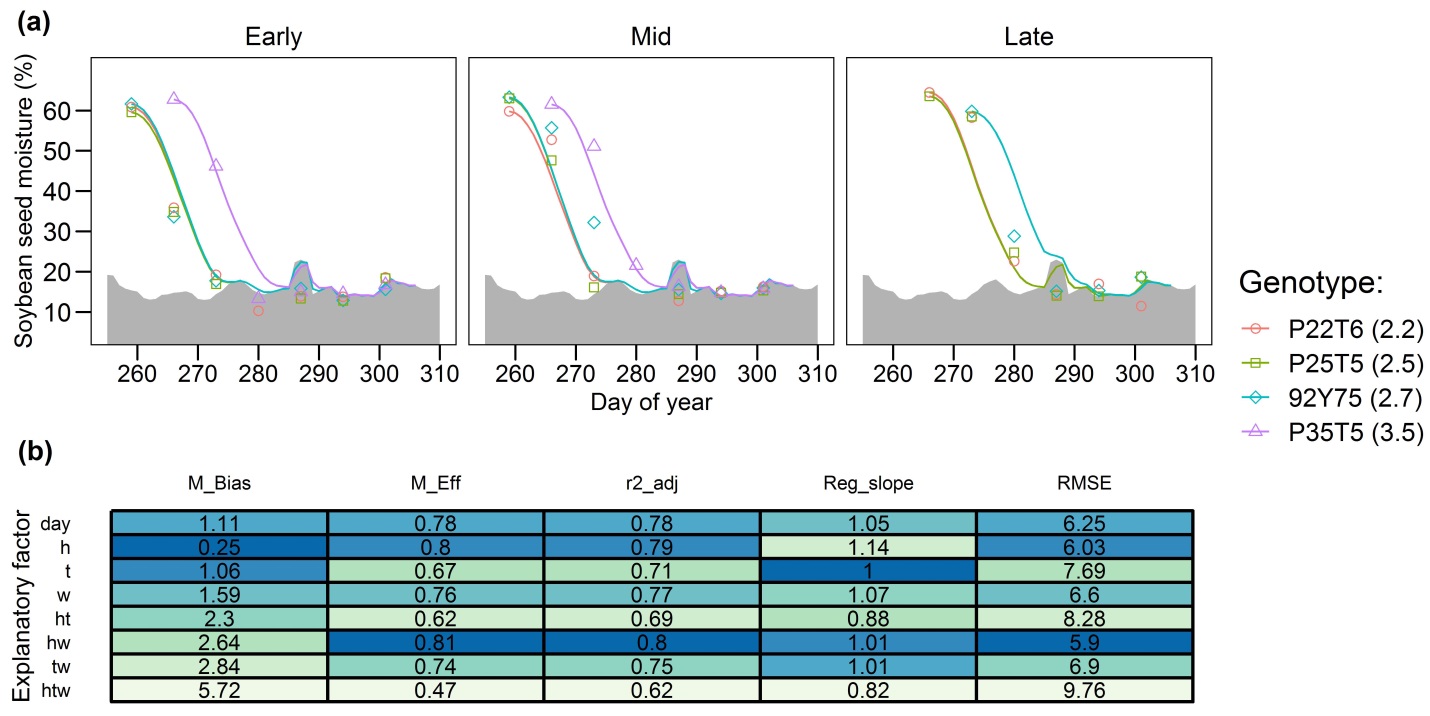


**Supplemental Figure S5**. (a) Implementation of the soybean grain dry-down across independent environmental conditions (Early, mid and late planting dates in 2014; testing dataset in Table 1), using values of relative humidity estimated from daily minimum and maximum temperatures. Solid lines represent simulation with the *day* algorithm, symbols represent the measured data, and shaded area represents the 3-day moving average equilibrium moisture content (*Me*). Numbers within parentheses next to the genotype name indicate cultivar relative maturity. (b) Model fit among all the explored algorithms is compared using the model bias (M_Bias), modeling efficiency (M_Eff), adjusted coefficient of determination (r2_adj), slope of the regression of measured vs predicted (Reg_slope) and root mean square error (RMSE). Dark blue shading indicates better fit.
